# Glycoprotein B switches conformation during murid herpesvirus 4 entry

**DOI:** 10.1099/vir.0.83519-0

**Published:** 2008-06

**Authors:** Laurent Gillet, Susanna Colaco, Philip G. Stevenson

**Affiliations:** Division of Virology, Department of Pathology, University of Cambridge, UK

## Abstract

Herpesviruses are ancient pathogens that infect all vertebrates. The most conserved component of their entry machinery is glycoprotein B (gB), yet how gB functions is unclear. A striking feature of the murid herpesvirus 4 (MuHV-4) gB is its resistance to neutralization. Here, we show by direct visualization of infected cells that the MuHV-4 gB changes its conformation between extracellular virions and those in late endosomes, where capsids are released. Specifically, epitopes on its N-terminal cell-binding domain become inaccessible, whilst non-N-terminal epitopes are revealed, consistent with structural changes reported for the vesicular stomatitis virus glycoprotein G. Inhibitors of endosomal acidification blocked the gB conformation switch. They also blocked capsid release and the establishment of infection, implying that the gB switch is a key step in entry. Neutralizing antibodies could only partially inhibit the switch. Their need to engage a less vulnerable, upstream form of gB, because its fusion form is revealed only in endosomes, helps to explain why gB-directed MuHV-4 neutralization is so difficult.

## INTRODUCTION

Herpesviruses are ubiquitous, persistent parasites whose behaviour impinges significantly on vertebrate biology. They characteristically use immune evasion to spread from primed, immunocompetent hosts, and viral CD8^+^ T-cell-evasion mechanisms are well known (Yewdell & Hill, 2002). Much less is known of how herpesviruses evade pre-formed antibody. We are using murid herpesvirus 4 (MuHV-4) to define molecular mechanisms behind the epidemiologically evident resistance of herpesviruses to neutralization (Xu *et al.*, 1996). One important factor may be antibody-coated virions exploiting host Fc receptors for uptake when normal cell binding is blocked (Rosa *et al.*, 2007). This critically requires that viral membrane fusion remains intact. Thus, a key question is how membrane fusion avoids inhibition by antibody.

One possibility is that the fusion machinery remains hidden on cell-free virions, much as conformation changes in the human immunodeficiency virus gp120 restrict antibody access until after cell binding (Chen *et al.*, 2005). Herpes simplex virus (HSV) entry is initiated by conformation changes in glycoprotein D (gD) (Fusco *et al.*, 2005; Krummenacher *et al.*, 2005), an alphaherpesvirus-specific addition to the core fusion complex (Spear & Longnecker, 2003). The inhibitory effects of gp150 on MuHV-4 infection of cells with low glycosaminoglycan (GAG) expression (de Lima *et al.*, 2004) and of gp350 on Epstein–Barr virus infection of epithelial cells (Shannon-Lowe *et al.*, 2006) suggest that gammaherpesvirus entry may be triggered similarly. However, gD, gp150 or gp350 could hardly protect the whole, multi-protein entry machinery. Their engagement is probably just the first of several conformation changes in virion glycoproteins that cumulate in membrane fusion. Understanding how each glycoprotein changes in the context of infection should tell us the limits imposed on antibody-mediated neutralization.

We have focused on gB, the most conserved component of herpesvirus membrane fusion (Turner *et al.*, 1998). The HSV gB structure (Heldwein *et al.*, 2006) provides a template for understanding these proteins as a whole. Comparison with vesicular stomatitis virus glycoprotein G (VSV-G) (Roche *et al.*, 2006, 2007) suggests that herpesvirus gBs might adopt distinct conformations during entry, with the solved structure a downstream form.

Although gB is exposed on MuHV-4 virions (Lopes *et al.*, 2004), it presents a very difficult neutralization target (Gillet *et al.*, 2006). As with other herpesviruses (Ohlin *et al.*, 1993; Holloway *et al.*, 1998; Akula *et al.*, 2002; Okazaki *et al.*, 2006), the gB N terminus is a neutralization target for MuHV-4 (Gillet *et al.*, 2006). However, this neutralization requires IgM monoclonal antibodies (mAbs), which are rare in MuHV-4 carriers, and even then remains incomplete. In order to understand how gB is exposed to antibody, we used conformation-specific mAbs to track its antigenicity during viral entry. By keeping to the context of infectious virions, we preserved important interactions between gB and other virion glycoproteins such as gH (Gillet & Stevenson, 2007a). We found evidence of a dramatic gB conformation shift that sheds new light on how herpesviruses resist neutralization.

## METHODS

### Cells and viruses.

BHK-21 fibroblasts, NMuMG epithelial cells, NIH-3T3 fibroblasts, 293T cells, NS0 myeloma cells, MCCD polarized murine epithelial cells, COS-7 cells, CHO-K1 cells (ATCC) and the gB–glycosylphosphatidylinositol (GPI)-expressing derivative CHO-gB (Lopes *et al.*, 2004) were all grown in Dulbecco's modified Eagle's medium (Invitrogen) supplemented with 2 mM glutamine, 100 U penicillin ml^−1^, 100 μg streptomycin ml^−1^ and 10 % fetal calf serum (PAA Laboratories). 293T cells were transfected with the GPI-linked gB extracellular domain or domains derived from it (Gillet *et al.*, 2006) by using FuGENE 6 (Roche Diagnostics). All viruses were derived from a cloned MuHV-4 bacterial artificial chromosome (Adler *et al.*, 2000). Virions were harvested from infected BHK-21 cell supernatants by ultracentrifugation; infected-cell debris was removed by low-speed centrifugation (May *et al.*, 2005a). Of note, rigorous removal of infected-cell debris was critical for clean immunofluorescence data. In some experiments, we purified virions further on Ficoll gradients, but this made no difference to the data obtained.

### mAbs.

mAbs were derived from MuHV-4-infected BALB/c mice at least 3 months post-infection by fusion of spleen cells with NS0 cells (Köhler & Milstein, 1975). The mAbs used in this study are listed in Table 1. All glycoprotein-specific mAbs were selected first by their capacity to recognize virus-infected cells; each was then typed for its target glycoprotein (Gillet *et al.*, 2007a).

### Neutralization assays.

Viruses were pre-incubated (2 h at 37 °C) with dilutions of immune sera or mAbs, and then added to BHK-21 or NMuMG cell monolayers. After a further 2 h, the monolayers were overlaid with 0.3 % carboxymethylcellulose. The monolayers were fixed in 4 % formaldehyde after 4 days for BHK-21 cells and after 6 days for NMuMG cells. The fixed cells were stained with 0.1 % toluidine blue and plaques were counted with a plate microscope (Olympus).

### Immunofluorescence.

Cells were plated onto coverslips overnight, then exposed to MuHV-4 virions (3 p.f.u. per cell). After three washes in PBS to remove unbound virions, the cells were fixed in PBS with 4 % paraformaldehyde (30 min) and permeabilized with 0.1 % Triton X-100 (15 min). Viral glycoproteins were detected with murine mAbs plus either Alexa 488- or Alexa 568-conjugated goat anti-mouse IgG (Invitrogen) or a combination of Alexa 488- or Alexa 633-conjugated goat anti-mouse IgG1 and Alexa 568-conjugated goat anti-mouse IgG2a. None of the MuHV-4 mAbs used for immunofluorescence gave detectable staining of uninfected cells. Lysosome-associated membrane protein 1 (LAMP-1) was detected with the rat mAb 104B (BD Pharmingen) and Alexa 488- or Alexa 568-conjugated goat anti-rat IgG (Invitrogen). Nuclei were counterstained with DAPI (4,6-diamidino-2-phenylindole). Fluorescence was visualized with a Leica confocal microscope imaging single 1 μm sections, except for Figs 2 and 3, when we used an Olympus IX70 microscope plus a Retiga 2000R camera line (QImaging).

### Flow cytometry.

Cells exposed to enhanced green fluorescent protein (eGFP)^+^ viruses were washed twice in PBS and analysed directly for green-channel fluorescence. For surface staining, cells were incubated (1 h at 4 °C) with MuHV-4 glycoprotein-specific mAbs followed by fluorescein-conjugated rabbit anti-mouse IgG pAb (Dako Cytomation). All cells were washed twice in PBS after treatment with each antibody and analysed on a FACScalibur (BD Biosciences).

## RESULTS

### Recombinant and infected-cell gB express the same epitopes, but in different proportions

**Fig. 1. f1:**
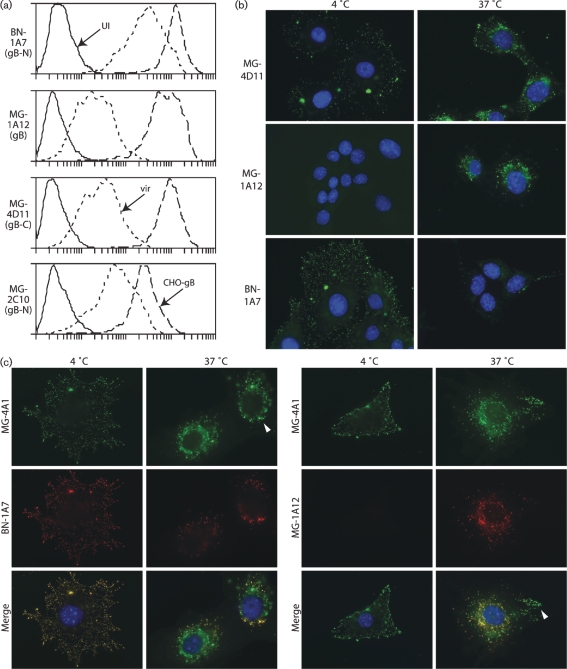
gB changes conformation after endocytosis. (a) mAbs were compared by flow cytometry for their staining of uninfected (UI, solid lines) or MuHV-4-infected (vir, short-dashed lines) BHK-21 cells, or CHO cells expressing a GPI-linked gB extracellular domain (CHO-gB, long-dashed lines). Each mAb is representative of at least five examples, each tested in at least three different experiments. (b) Virions were attached to NMuMG cells (2 h at 4 °C), washed three times with PBS, then either fixed immediately (4 °C) or first incubated (2 h at 37 °C) to allow endocytosis. All cells were then permeabilized and stained for gB (green). Nuclei were counterstained with DAPI (blue). Equivalent data were obtained in five repeat experiments. (c) Cells were incubated with virions (2 h at 4 °C), with or without allowing subsequent endocytosis (2 h at 37 °C) as in (b), then fixed, permeabilized and stained with the gB-specific mAbs BN-1A7 or MG-1A12 (both IgG2a, red), plus MG-4A1 (IgG1, green), which recognizes an invariant epitope. Co-localization of isotype-specific secondary-antibody staining is yellow in the merged image. The arrowhead in the MG-4A1/BN-1A7 merge shows co-localization outside more central MG-4A1-only staining. The arrowhead in the MG-4A1/MG-1A12 merge shows peripheral MG-4A1-only staining, with more central co-localization. Equivalent data were obtained in three repeat experiments.

**Fig. 2. f2:**
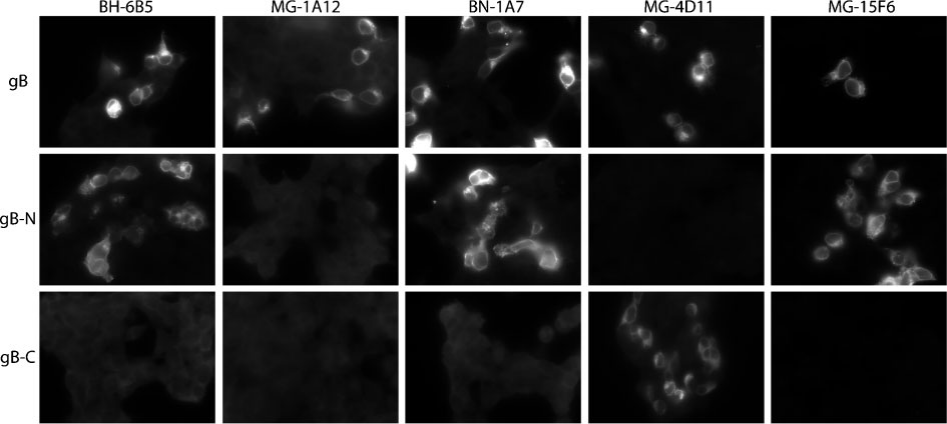
Localization of mAb epitopes on gB. In order to map mAb recognition, 293T cells were transfected with the full-length gB extracellular domain fused to a GPI membrane anchor (gB), or with GPI-linked fragments of this domain either N-terminal (gB-N) or C-terminal (gB-C) to its furin-cleavage site (Lopes *et al.*, 2004). For gB-C expression, the native gB signal sequence was retained as described previously (Gillet *et al.*, 2006). Forty-eight hours after transfection, each population was fixed, permeabilized and stained with gB-specific mAbs as indicated. mAb MG-2C10 gives high background intracellular staining; we have therefore shown MG-15F6 for comparison, an IgG whose recognition site maps very close to that of MG-2C10 at the gB N terminus (see also Fig. 7c[Fig f7]). The neutralizing mAb BH-6B5 is also shown for comparison.

mAbs that recognize both infected cells and recombinant gB define its accessible surface (Lopes *et al.*, 2004). The relative efficiencies with which such mAbs recognized each form of gB varied (Fig. 1a[Fig f1]). MG-2C10, a neutralizing mAb that recognizes a linear epitope near the MuHV-4 gB N terminus (Gillet *et al.*, 2006), showed less difference between native and recombinant gB than did MG-4D11, which recognizes an epitope C-terminal to the gB furin-cleavage site (gB-C). BN-1A7 recognizes an epitope N-terminal to the gB furin-cleavage site (gB-N) (Fig. 2) and was similar to MG-2C10. MG-1A12, which requires both gB-N and gB-C for recognition, was similar to MG-4D11. Thus, native and recombinant gB were antigenically distinct. BHK-21 cells infected with vaccinia virus expressing gB–GPI (Gillet *et al.*, 2007b) showed staining similar to that of CHO-gB cells (data not shown). The BN-1A7 and MG-1A12 staining patterns were each observed with at least eight different gB-specific mAbs. All of those mapping to gB-N were similar to BN-1A7.

### Virion gB changes its antigenicity during virus entry

Infected cells may display antigens from entering and exiting virions and non-virion glycoproteins. We analysed entering virions specifically by binding them to uninfected cells. MuHV-4 infects via endocytosis (Gill *et al.*, 2006), so we first bound the virions at 4 °C and then shifted the temperature to 37 °C to compare pre- and post-endocytosis (Fig. 1b[Fig f1]). Single cells are shown for optimal resolution. In Fig. 1, as in subsequent figures, each cell shown is fully representative of at least 75 % of the total examined (*n*>100).

**Fig. 3. f3:**
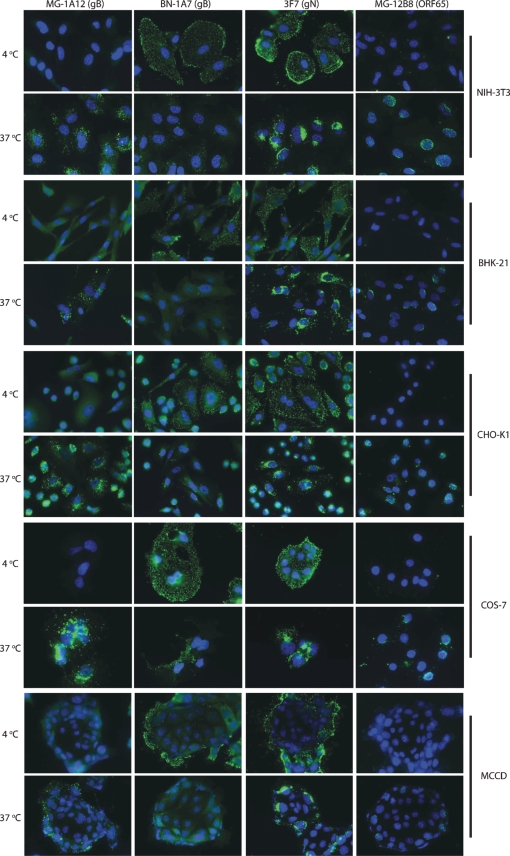
Different cell lines show the same gB conformation change. Wild-type MuHV-4 virions were bound to cells (2 h at 4 °C). Unbound virions were then removed by washing with PBS and the cells were either fixed immediately (4 °C) or after a further incubation (2 h at 37 °C) to allow endocytosis. All cells were then permeabilized with 0.1 % Triton X-100 and stained for MuHV-4 virion components as shown (green). Nuclei were counterstained with DAPI (blue). The MG-12B8 capsid epitope only becomes accessible after virion uncoating (Gillet *et al.*, 2006). The gN epitope is always accessible. In each cell line, gB switched from BN-1A7^+^ before endocytosis to MG-1A12^+^ after endocytosis.

**Fig. 4. f4:**
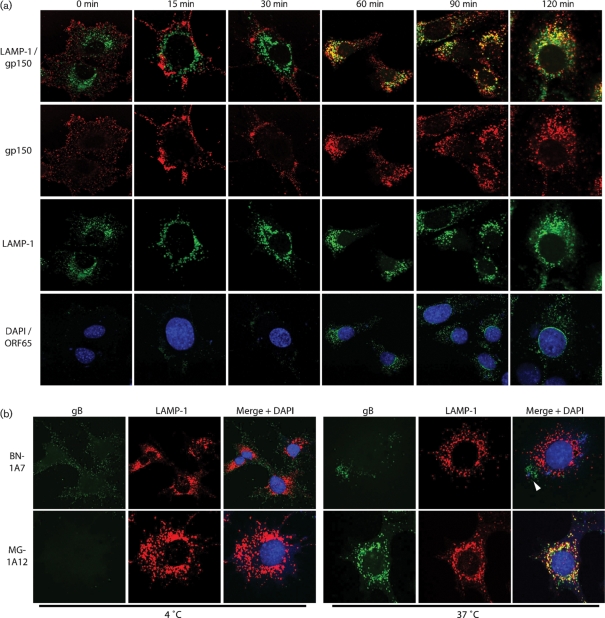
Glycoprotein conformation changes coincide with capsid release. (a) Virions were incubated with NMuMG cells (2 h at 4 °C), unbound virions were removed by washing three times with PBS and the cells were incubated at 37 °C for the time indicated before fixing with paraformaldehyde, permeabilizing with Triton X-100 and staining for the ORF65 capsid component with MG-12B8 [Alexa 568 (shown as green), with DAPI nuclear counterstaining in blue]. The cells were also stained for LAMP-1 (Alexa 488, green) and for gp150 with mAb BN-3A4 (Alexa 633, red). LAMP-1/gp150 co-localization appears yellow. Equivalent results were obtained in four further experiments. (b) NMuMG cells were exposed to virions (2 h at 4 °C), then washed three times with PBS and either fixed immediately or first incubated for 2 h at 37 °C. All cells were then permeabilized and stained for LAMP-1 (red) and for gB with mAbs BN-1A7 or MG-1A12 (green). Co-localization appears yellow. The arrowhead indicates residual BN-1A7 staining confined to LAMP-1^−^ endosomes. Equivalent data were obtained in two further experiments.

mAb MG-4D11 recognized gB both before and after endocytosis. In contrast, BN-1A7 recognized gB strongly at the cell surface and poorly after endocytosis, whilst MG-1A12 recognized endocytosed gB only. Virion gB therefore changed its antigenicity during entry into NMuMG cells. Other cell lines showed the same change (Fig. 3). It occurred after endocytosis, as BN-1A7 staining still co-localized with an invariant epitope defined by mAb MG-4A1 in peripheral endosomes, whereas MG-1A12 staining co-localized with MG-4A1 only in more central endosomes (Fig. 1c[Fig f1]; see also Fig. 4b[Fig f4]). This difference in co-localization made it clear that BN-1A7 and MG-1A12 recognize gB at different stages of entry. The complete shift in virion gB during entry from BN-1A7^+^MG-1A12^−^ to BN-1A7^−^MG-1A12^+^ argued that these forms are mutually exclusive. Thus, gB-transfected and MuHV-4-infected cells, which were each recognized by both mAbs, must each express both forms, but in different proportions.

The MuHV-4 gB-N corresponds to domains I and II of the HSV gB, which hang down on extended peptide loops in its published structure (Heldwein *et al.*, 2006). The domain II equivalent of the structurally analogous VSV-G (its domain III) occupies a comparable position in its post-fusion conformation (Roche *et al.*, 2006), but is more exposed in the pre-fusion form, where it contributes much of the likely receptor-binding site (Roche *et al.*, 2007). A similar pre-fusion exposure of gB domain II would be consistent with the cell binding of soluble gB-N (Gillet *et al.*, 2007c) and with gB-N-specific mAbs, such as BN-1A7, preferentially recognizing pre-fusion gB (Fig. 1); the post-endocytic loss of BN-1A7 staining would reflect domain II moving to a more dependent position in post-fusion gB. It therefore seemed likely that gB has pre- and post-fusion conformations, much like VSV-G (we keep to the convention of ‘post-fusion’ being the fusion-competent form of a glycoprotein, although the VSV-G and gB crystal structures include neither ligand nor lipid and so are not strictly post-fusion). The main alternative explanation for one gB conformational epitope disppearing at the same time as another appears – an elaborate shift in an antigen-masking protein from one site on gB to another – seems very unlikely. The only MuHV-4 glycoproteins known to associate with gB are gH/gL and gp150 (Gillet & Stevenson, 2007a). Virions lacking gL, which display a markedly different gH conformation from the wild type (Gillet *et al.*, 2007d), or lacking gp150 showed exactly the same changes in gB antigenicity (data not shown).

### The gB conformation change occurs close to membrane fusion

How does the gB conformation change relate to membrane fusion? mAb MG-12B8 defines an MuHV-4 capsid epitope that is inaccessible on intact virions, but revealed once they have uncoated (Gillet *et al.*, 2006). The MG-12B8 epitope appears when incoming virion glycoproteins reach the late endosomes/lysosomes marked by LAMP-1 (Marsh *et al.*, 1987) (Fig. 4a[Fig f4]). This coincides with the change in gB antigenicity (Fig. 4b[Fig f4]): MG-1A12 staining co-localized with LAMP-1, whereas BN-1A7 staining was evident only in peripheral, LAMP-1^−^ endosomes. The gB conformation change therefore occurs close in time and place to membrane fusion. This implied that BN-1A7^+^ gB engages in binding, whilst MG-1A12^+^ gB mediates fusion.

### The gB conformation change is pH-dependent

**Fig. 5. f5:**
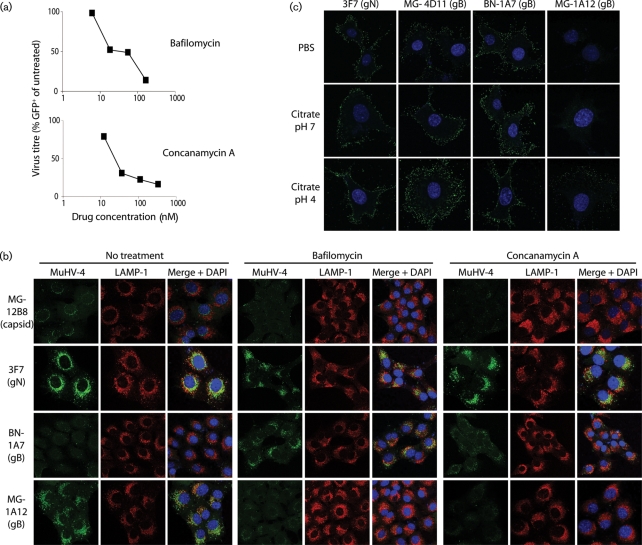
The gB conformation change is pH-dependent. (a) NMuMG cells were left untreated or cultured with bafilomycin or concanamycin A as shown, and exposed to MuHV-4 expressing eGFP under the control of a cytomegalovirus IE-1 promoter. Eighteen hours later, the proportion of eGFP^+^ cells was quantified by flow cytometry. Equivalent data were obtained in two further experiments. The same drug concentrations had no effect on infection by HSV (data not shown). (b) MuHV-4 virions were bound to the cells (2 h at 4 °C) in the presence or absence of drugs as shown. The cells were then washed in PBS, incubated with or without 100 nM bafilomycin or 100 nM concanamycin A for a further 2 h at 37 °C, then fixed, permeabilized and stained for viral proteins. Equivalent data were obtained in two further experiments. (c) Virions were bound to NMuMG cells (2 h at 4 °C), then exposed to different pH buffers (15 min at 37 °C), fixed, permeabilized and stained for virion glycoproteins as shown. Equivalent data were obtained in two further experiments.

**Fig. 6. f6:**
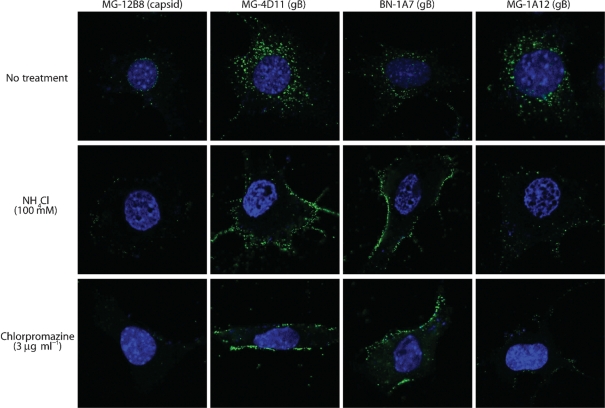
Blocking endocytosis also blocks the gB conformation change. NMuMG cells were treated with NH_4_Cl or chlorpromazine as shown and exposed to wild-type MuHV-68 virions (4 °C for 2 h, then washed three times in PBS, then 37 °C for 2 h). The cells were then fixed, permeabilized and stained for MuHV-68 virion components as shown (green). Nuclei were counterstained with DAPI (blue). Representative cells are shown. Both drug treatments inhibited the shift in gB from BN-1A7^+^ to MG-1A12^+^. Although NH_4_Cl is classically an inhibitor of endosomal acidification, it evidently acted here mainly by blocking virion endocytosis.

The delay in MuHV-4 capsid release until virions reach LAMP-1^+^ endosomes (Fig. 4a[Fig f4]) suggests that membrane fusion requires a low pH. This was confirmed by inhibitors of lysosomal acidification blocking both infection (Fig. 5a[Fig f5]) and capsid release (Fig. 5b[Fig f5]). Virion glycoproteins (gN in Fig. 5b[Fig f5]) were still endocytosed and reached LAMP-1^+^ endosomes, but the BN-1A7 gB epitope was preserved and now co-localized with LAMP-1, whereas the MG-1A12 epitope failed to appear. Thus, the gB conformation change was pH-dependent and again linked to membrane fusion. NH_4_Cl also blocked the conformation change but, like chlorpromazine, mainly blocked endocytosis (Fig. 6).

Exposing cell-bound virions to low pH triggered the gB conformation change with only low efficiency (Fig. 5c[Fig f5]). Thus, exposure to a pH of 4 increased MG-1A12 gB staining, but without much change in BN-1A7 staining. The number of gB molecules changing was evidently much lower than in normal infection (Fig. 1b[Fig f1]). This argues that ligand engagement or another aspect of the endosomal environment contributes to the gB switch. Whilst not necessarily sufficient, low pH was clearly necessary.

### Neutralization is limited to IgMs that recognize gB-N

**Fig. 7. f7:**
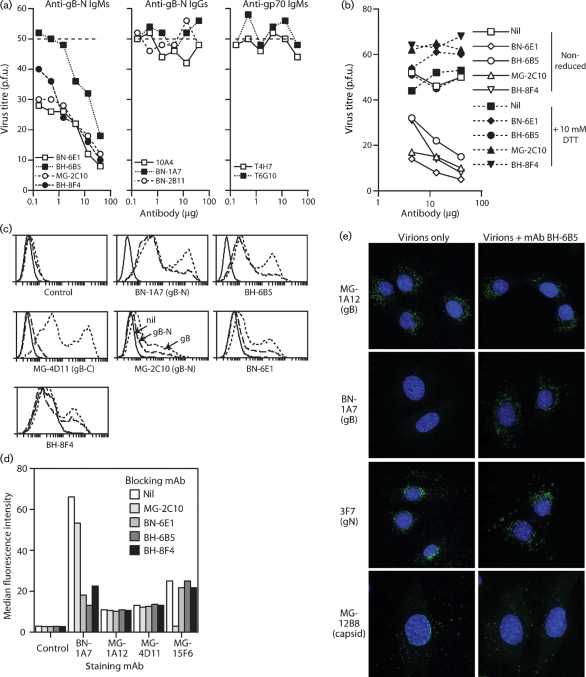
Neutralizing mAbs struggle to block the gB conformation change. (a) MuHV-4 virions were incubated with antibody dilutions (2 h at 4 °C), then plaque-assayed on BHK-21 cells. Gp70 is the complement control protein encoded by ORF4. The horizontal dashed line shows plaque numbers with virus alone. Equivalent data were obtained in three repeat experiments. (b) The pentameric neutralizing IgMs were reduced to monomers with 10 mM dithiothreitol (DTT; 15 min at 4 °C), then tested for plaque reduction on BHK-21 cells as before. Nil, ±DTT without antibody. Equivalent data were obtained in one repeat experiment. All reduced IgMs still stained virus-infected cells by flow cytometry (data not shown). (c) 293T cells were transfected with empty vector (solid lines), gB–GPI (short dashes) or gB-N–GPI (long dashes), then trypsinized 48 h later and analysed for mAb recognition by flow cytometry. Control, secondary antibody only. All of the mAbs recognized gB–GPI. All of the neutralizing mAbs (MG-2C10, BN-6E1, BH-8F4 and BH-6B5) recognized gB-N. BN-1A7 is a gB-N-specific control; MG-4D11 is a gB-C-specific control. (d) MuHV-4-infected BHK-21 cells (2 p.f.u. per cell, 18 h) were incubated or not (nil) with gB-specific IgMs (30 μg ml^−1^, 1 h at 4 °C), then stained with gB-specific IgG2a mAbs (2 μg ml^−1^). Control, secondary antibody only. Thirty thousand cells per sample were then analysed by flow cytometry. The reductions in BN-1A7 staining by BN-6E1, BH-6B5 and BH-8F4 were highly significant (*P*<0.0001 by Student's *t*-test), as was the reduction in MG-15F6 staining by MG-2C10 (*P*<0.0001). (e) MuHV-4 virions were pre-incubated or not with mAb BH-6B5, then added to NMuMG cells (2 h at 4 °C). The cells were then washed three times with PBS and incubated further (2 h at 37 °C) to allow endocytosis. The cells were then fixed, permeabilized and stained with IgG2a mAbs plus an IgG2a-specific secondary antibody as shown. Similar results were obtained in two repeat experiments and with the other neutralizing IgMs.

MG-2C10 is representative of several IgM mAbs that recognize the gB N terminus and block infection at a post-binding step (Gillet *et al.*, 2006). In three further fusions, we identified three more gB-specific neutralizing mAbs (in addition to re-isolating MG-2C10-like mAbs) (Fig. 7a[Fig f7]). Again, all were IgMs. None of 85 gB-specific IgGs identified in the same fusions gave significant neutralization; Fig. 7(a) shows three examples. mAb reduction confirmed that a pentameric structure was crucial for neutralization (Fig. 7b[Fig f7]). Neutralization was not a general property of IgMs, as gp70-specific IgMs failed to neutralize (Fig. 7a[Fig f7]).

Although most gB-specific mAbs were like MG-1A12 and required all of gB for recognition, the gB-specific neutralizing IgMs all mapped to gB-N (Fig. 7c[Fig f7]). The new IgMs recognized a different epitope than MG-2C10. Thus, they reduced BN-1A7 binding much more than MG-2C10 did and had no effect on MG-15F6 binding, whereas MG-2C10 inhibited MG-15F6 markedly (Fig. 7d[Fig f7]) (based on transfected gB truncation mutants, MG-15F6 recognition requires residues 13–30 after the predicted gB signal sequence cleavage, but not residues 2–13; MG-2C10 requires residues 2–6). The failure of gB-N-specific IgGs such as BN-1A7 and MG-15F6 to neutralize, even though their recognition sites overlapped with those of neutralizing IgMs, emphasized further that neutralization requires an IgM isotype. Indeed, the more abundant gB-N-specific IgGs in immune sera could conceivably outcompete gB-N-specific IgMs to impair neutralization.

### The gB conformation change is difficult for antibodies to block

As with MG-2C10 (Gillet *et al.*, 2006), BN-6E1, BH-6B5 and BH-8F4 blocked neither cell binding nor virion endocytosis. Fig. 7(e) shows data for BH-6B5; the other mAbs were equivalent. They partly inhibited the gB conformation switch – BH-6B5 was the most effective, retaining some BN-1A7^+^ gB. However, unlike bafilomycin or concanamycin A (Fig. 5b[Fig f5]), they failed to block it completely – Fig. 7(e) shows that MG-1A12^+^ gB still appeared. Thus, once virions get to late endosomes, the gB conformation switch seems to be hard for antibodies to block. As gB-N incorporates the putative gB fusion loops (Heldwein *et al.*, 2006), the gB-N-specific IgMs seemed more likely to neutralize by hindering fusion sterically. This would explain why they must be IgMs, as the much larger size of IgMs would vastly increase their scope for steric hindrance.

## DISCUSSION

The MuHV-4 gB is a major component of virions (Lopes *et al.*, 2004). We have shown here that gB changes its antigenicity during viral entry, consistent with a shift between cell-binding and pro-fusion conformations. The gB N-terminal domains, which participate in cell binding (Gillet *et al.*, 2007c), were more accessible on pre-fusion gB. Their reduced accessibility after endocytosis was consistent with the conformation change described for VSV-G, with the solved gB structure (Heldwein *et al.*, 2006) being its ‘post-fusion’ form. The gB conformation switch probably precedes actual fusion, as GPI-linked gB readily adopted the ‘post-fusion’ form.

Low pH was important for the gB conformation switch. This may explain why the MuHV-4 gB is a poor neutralization target (Gillet *et al.*, 2006). A requirement for low pH would also explain why MuHV-4, like Kaposi's sarcoma-associated herpesvirus (KSHV) (Akula *et al.*, 2003), fails to fuse with plasma membranes. Plasma-membrane fusion is possible with transfected KSHV glycoproteins (Pertel, 2002), and transfected MuHV-4 gB could adopt its pro-fusion form (Fig. 1a[Fig f1]). However, this is a very different setting from infection. In the absence of other virion glycoproteins, transfected gB may switch its conformation before reaching the cell surface, perhaps in the acidic environment of the trans-Golgi network (Demaurex *et al.*, 1998).

Although the cell-binding gB N-terminal domains are accessible on MuHV-4 virions, these virions readily escape from infected cells, suggesting that the binding site itself is hidden. Moreover, gB-N–Fc binds to a non-GAG ligand, but MuHV-4 cell binding is highly GAG-dependent (Gillet *et al.*, 2007c). gp150 regulates MuHV-4 binding to a non-GAG cellular ligand (de Lima *et al.*, 2004; Gillet *et al.*, 2007c), as gp150 knockouts are much less GAG-dependent than the wild type (also, virions lacking gp150 do have a problem escaping from infected cells). The obvious explanation would be that gp150 covers the gB-N binding epitope until displaced by GAGs. Thus, gB has 2-fold protection: masking by gp150 and a post-endocytic conformation change. This protection allows efficient virion release and hides gB from antibody.

Many mechanisms of virion neutralization have been described (Klasse & Sattentau, 2002). By far the most common and certainly the most clearly defined is a block to receptor binding (Knossow & Skehel, 2006). However, the capacity of herpesviruses such as MuHV-4 to escape from endosomes and infect Fc receptor-positive cells productively (Rosa *et al.*, 2007) forces neutralization to act at post-binding steps, such as membrane fusion. Such neutralization must cope with the dramatic conformation changes characteristic of viral fusion proteins (Knossow & Skehel, 2006). If antibody can bind only to the upstream form of a glycoprotein, because its switch is secluded, neutralization will be limited to steric hindrance – binding common epitopes close to the fusion loops if such exist – or to blocking the switch itself. Blocking the gB conformation switch was evidently hard, presumably because the pre-fusion conformation becomes highly unstable in late endosomes. Also, steric hindrance seems to require an IgM isotype, which is rare in steady-state B-cell responses. The gB conformation change therefore helps MuHV-4 to evade neutralization.

A post-endocytic gB conformation switch readily explains why gB-directed MuHV-4 neutralization is more difficult than for HSV (Bender *et al.*, 2007) or human cytomegalovirus (Speckner *et al.*, 1999). In the typical setting of neutralization assays, HSV and cytomegalovirus fuse at the plasma membrane (Compton *et al.*, 1992; Browne *et al.*, 2001); conformation-switched gB is therefore accessible. Neutralizing mAbs have accordingly been mapped to prominent positions on post-fusion gB (Speckner *et al.*, 1999; Heldwein *et al.*, 2006; Bender *et al.*, 2007). In contrast, mAbs specific for post-fusion MuHV-4 gB, such as MG-1A12, never neutralized. HSV and cytomegalovirus can also infect via endocytosis (Clement *et al.*, 2006; Ryckman *et al.*, 2006) and this route may be harder for gB-specific mAbs to block. However, a more important consideration is where neutralization might act in the viral life cycle. Herpesviruses mostly spread within their hosts via cell–cell contacts (Peeters *et al.*, 1993; Dingwell *et al.*, 1994). These probably exclude antibody (Roth & Compans, 1980). Thus, antibody reduces the intra-host lytic spread of HSV, mainly by Fc receptor-dependent effector functions (Balachandran *et al.*, 1982; Rector *et al.*, 1982; Kohl *et al.*, 1986); Fc inhibition, in turn, increases intra-host spread (Nagashunmugam *et al.*, 1998). Neutralization is more relevant to cell-free virions, whose key role is inter-host spread. Virions are exposed to antibody when they exit an immune host. However, once they reach a new, naive host, the only antibody capable of blocking infection is that already attached. Epitopes revealed by conformation changes are therefore safe: as with endocytic uptake, they are effectively secluded from pre-formed antibody. Once transmission has occurred, cell–cell spread again takes over; the window for effective neutralization is shut.

Fusion-complex conformation changes do not act alone in antibody evasion. The highly immunogenic gp150 both drives Fc receptor-dependent infection and, through its immunodominance, suppresses the production of antibodies that might otherwise neutralize (Gillet *et al.*, 2007a). The gB N terminus is protected by *O*-glycosylation and, in turn, protects a key epitope on gH/gL (Gillet & Stevenson, 2007b). Such mechanisms complement that described here by reducing the exposure of upstream forms of virion fusion proteins to antibody. For MuHV-4 at least, much of the complexity of herpesvirus entry makes sense as antibody evasion.

## Figures and Tables

**Table 1. t1:** mAbs used in this study

**mAb**	Target*	**Isotype**	**Epitope†**	**Reference**
BN-1A7	gB-N	IgG2a	Conformational	This paper
BN-2B11	gB-N	IgG2a	Conformational	This paper
10A4	gB-N	IgG2a	Conformational	This paper
MG-15F6	gB-N	IgG2a	Conformational	This paper
BH-6B5	gB-N	IgM	Conformational	This paper
MG-2C10	gB-N	IgM	Linear	Gillet *et al.* (2006)
BN-6E1	gB-N	IgM	Linear	This paper
BH-8F4	gB-N	IgM	Linear	This paper
MG-1A12	gB	IgG2a	Conformational	Gillet *et al.* (2006)
MG-4A1	gB	IgG1	Conformational	This paper
MG-4D11	gB-C	IgG2a	Linear	Gillet *et al.* (2006)
T4H7	gp70	IgM	Conformational	This paper
T6G10	gp70	IgM	Conformational	This paper
BN-3A4	gp150	IgG1	Linear	This paper
3F7	gN	IgG2a	Linear	May *et al.* (2005b)
MG-12B8	ORF65 (capsid)	IgG2a	Linear	Gillet *et al.* (2006)

*gB-N, The portion of gB N-terminal to its furin-cleavage site was sufficient for mAb recognition; gB-C, the portion C-terminal to the furin-cleavage site. †Based on the recognition or not of denatured protein in immunoblots.
